# A simple rocker‐induced mechanical stimulus upregulates mineralization by human osteoprogenitor cells in fibrous scaffolds

**DOI:** 10.1002/term.2462

**Published:** 2017-08-09

**Authors:** Sasima Puwanun, Robin M. Delaine‐Smith, Helen E. Colley, Julian M. Yates, Sheila MacNeil, Gwendolen C. Reilly

**Affiliations:** ^1^ Faculty of Dentistry Naresuan University Thailand; ^2^ Department of Materials Science and Engineering University of Sheffield UK; ^3^ Barts Cancer Institute, Queen Mary University of London UK; ^4^ School of Clinical Dentistry University of Sheffield UK; ^5^ Oral and Maxillofacial Surgery and Implantology, School of Dentistry University of Manchester UK; ^6^ INSIGNEO Institute for in silico Medicine University of Sheffield UK

**Keywords:** cleft palate, electrospinning, fluid flow, human jaw periosteal cells, marrow stromal cells, osteogenesis

## Abstract

Biodegradable electrospun polycaprolactone scaffolds can be used to support bone‐forming cells and could fill a thin bony defect, such as in cleft palate. Oscillatory fluid flow has been shown to stimulate bone production in human progenitor cells in monolayer culture. The aim of this study was to examine whether bone matrix production by primary human mesenchymal stem cells from bone marrow or jaw periosteal tissue could be stimulated using oscillatory fluid flow supplied by a standard see‐saw rocker. This was investigated for cells in two‐dimensional culture and within electrospun polycaprolactone scaffolds. From day 4 of culture onwards, samples were rocked at 45 cycles/min for 1 h/day, 5 days/week (rocking group). Cell viability, calcium deposition, collagen production, alkaline phosphatase activity and vascular endothelial growth factor secretion were evaluated to assess the ability of the cells to undergo bone differentiation and induce vascularisation. Both cell types produced more mineralized tissue when subjected to rocking and supplemented with dexamethasone. Mesenchymal progenitors and primary human mesenchymal stem cells from bone marrow in three‐dimensional scaffolds upregulated mineral deposition after rocking culture as assessed by micro‐computed tomography and alizarin red staining. Interestingly, vascular endothelial growth factor secretion, which has previously been shown to be mechanically sensitive, was not altered by rocking in this system and was inhibited by dexamethasone. Rocker culture may be a cost effective, simple pretreatment for bone tissue engineering for small defects such as cleft palate.

## 1 INTRODUCTION

Cleft lip and/or cleft palate is a common oral and facial malformation birth defect with a worldwide incidence of approximately 1.7 per 1000 live births (Mossey, Little, Munger, Dixon, & Shaw, 2009). The current treatment necessitates multiple operations over several years and involves bone harvesting from the iliac crest (hip bone), resulting in donor site morbidity. The alveolar ridge region is usually filled with an autologous bone graft to support permanent tooth eruption. Many researchers in the field have suggested investigating potential alternatives to bone graft as a treatment for the reconstruction of cleft palate (Lohberger et al., 2013). Moy, Lundgren, and Holmes (1993) found that bone graft substitute materials implanted alone instead of autologous bone graft resulted in a lower success rate compared with the combination of cells and bone graft substitute. This suggests that tissue engineering strategies using cells combined with a supportive biomaterial scaffold may be a more effective treatment for cleft palate repair than biomaterials alone (Behnia, Khojasteh, Soleimani, Tehranchi, & Atashi, 2012; Khojasteh, Eslaminejad, & Nazarian, 2008).

Mesenchymal stem cells (MSC) have been widely used for bone regeneration due to their ability to self‐renew and differentiate into osteoblastic cells, using specific culture media. Harvesting bone marrow MSC (hBMSC) from the iliac crest incurs less pain at the operation site, fewer arterial and nerve injuries, and a lower risk of infection compared with harvesting of traditional iliac crest bone grafts (Samee et al., 2008). Tissue engineering strategies using MSCs from patients have shown promise for reconstruction of a cleft defect at the alveolar ridge region; resulting in a successful repair and support of tooth eruption over the following 2 years (Hibi, Yamada, Ueda, & Endo, 2006). Another interesting cell source for bone repair is human periosteal cells. Periosteal cells reside in a connective tissue membrane that covers the outer surface of all bones, except at the joints of long bones, they can be extracted from human jaw periosteum (HJP) and appear to contain a subpopulation of MSCs. Studies have shown that HJP derived cells (HJPCs) show promise for bone tissue engineering due to reduced donor site morbidity and time of operation compared to autologous bone graft from iliac crest (Trautvetter, Kaps, Schmelzeisen, Sauerbier, & Sittinger, 2011). HJP tissue is easy to harvest during wisdom tooth removal or other routine maxillofacial surgical procedures. For patients with cleft palate, HJP tissue could be harvested from the palate at the time of palatal closure which typically takes place at age 6–12 months (National Health Service, 2010).

Polycaprolactone (PCL) electrospun scaffolds are a promising support for bone forming cells as they show low biodegradation rates, good mechanical properties (Jha et al., 2011) and PCL is approved for medical use by the US Food and Drug Administration (Hutmacher et al., 2001). Electrospinning is a straightforward fabrication method able to produce fibres of various diameters from 5 nm to several μm (Zargarian & Haddadi‐Asl, 2010). The nonwoven fibrous sheets formed are suitable for tissue engineering a thin region of bone, such as the palate.

Several studies have shown that fluid flow‐induced mechanical stress can enhance the osteogenic differentiation of MSCs, as previously reviewed (Delaine‐Smith, MacNeil, & Reilly, 2012; Delaine‐Smith, Sittichokechaiwut, & Reilly, 2014). Oscillatory fluid flow (OFF) is similar to the flow found in the canalicular system in mature bone and in the bone marrow (Gurkan & Akkus, 2008). Previous work, demonstrated that OFF applied using a standard see‐saw rocker stimulated mineralized matrix production by osteoblasts and embryonic derived osteoprogenitor cells in monolayer culture (Delaine‐Smith et al., 2012). Based on these findings it was hypothesized that OFF could increase osteogenic differentiation and mineralization of primary osteoprogenitor cells to precondition a construct for cleft palate patients. The aims of this study were firstly to determine if clinically applicable primary adult osteoprogenitor cells respond to rocker culture in a similar way to the cell lines previously examined and to determine if the angiogenic factor vascular endothelial growth factor (VEGF) was upregulated by OFF culture. Secondly, the mechanical stimulation technique was applied to a tissue engineered construct suitable for cleft palate: an electrospun PCL scaffold seeded with mesenchymal progenitor cells.

## 2 MATERIALS AND METHODS

All chemicals and culture consumables were obtained from Sigma–Aldrich (Poole, Dorset, UK) unless otherwise stated and used as supplied.

### 2.1 PCL scaffold fabrication and characterization

PCL pellets (molecular weight 80,000) were dissolved in dichloromethane (Fisher Scientific, Loughborough, UK) at a concentration of 10 wt%. Solutions were stirred for a minimum of 24 h at room temperature. Scaffolds were fabricated using an electrospinning rig as previously described (Bye et al., 2013; Delaine‐Smith, Green, Matcher, MacNeil, & Reilly, 2014). Sheets of PCL microfibers were fabricated at 17 kV, a flow rate of 40 μl/min, a working distance of 17 cm, and a drum rotation speed of 300 rotations/min (rpm) at room temperature. The solution was delivered via four needles to a rotating drum collector and the resulting scaffolds dried at room temperature and sterilized with 0.1% *v*/v peracetic acid before use.

Scaffold characterization was performed by scanning electron microscopy (SEM, Phillips XL‐20 SEM, Amsterdam, The Netherlands) of scaffolds coated with a gold ultrathin layer. Mean fibre diameter and their distribution were measured over 100 randomly selected fibres from four recorded SEM micrographs using image analysis software (ImageJ, National Institute of Health, USA). Each picture was randomly overlaid with a square grid size 2500 μm^2^ and 25 points were selected for fibre diameter measurements.

### 2.2 Cell culture

Three different cell types were used in this study: 1) primary HJPCs isolated from the periosteum tissue which was harvested from the jaw of patients undergoing maxillofacial surgery at the Charles Clifford Dental Hospital, Sheffield (with written, informed consent). This waste tissue collection was conducted under ethical approval 09/H1308/66 from the NRES Committee Yorkshire and The Humber, Sheffield; 2) human MSC (hBMSCs), isolated from bone marrow mononuclear cells from three individual donors (Lonza^®^, Castleford, UK); and 3) human embryonic cell derived mesenchymal progenitor cells hES‐MPs 002.5 (hESMP; Cellartis, Gothenburg, Sweden). A schematic diagram detailing which methods were used with each cell type can be found in Figure S1. HJPCs were not used for scaffold culture due to there not being sufficient numbers at low passages. hESMPs were the only cell type used for the micro‐computed tomography (μCT) study as they secreted more mineral on average, and therefore mineral deposition was more likely to be detectable by μCT.

All cell types were cultured in an expansion media (EM) which consisted of α‐minimal essential culture medium (Lonza^®^, Basel, Switzerland), supplemented with 10% fetal calf serum (*v*/v), 2 mm L‐glutamine, 100 mg/ml penicillin and streptomycin (P/S; basal culture medium). hESMPs were cultured in precoated 1% gelatine 75 cm^2^ tissue‐culture flasks. For osteogenesis induction media (OIM), EM was supplemented with 50 μg/ml ascorbic acid‐2‐phosphate and 5 mm β‐glycerolphosphate, or without the addition of dexamethasone (Dex), which was termed supplemented media (SM). Dex (10 nm) was added to HJPC and hBMSC cultures and 100 nm Dex to hESMP cultures based on previous experiments to establish the best Dex concentration for osteogenic induction. All cells were cultured at 37 °C in 5% CO_2_ in a humidified atmosphere. Media was changed every 2–3 days. HJPCs and hESMPs were used between passages 3 and 8, as suggested by (De Bari et al., 2006) and hBMSC cells were used between passages 2 and 3.

### 2.3 Cell isolation

HJPCs were isolated from freshly isolated periosteum tissue of two different donors referred to as HJPC‐1 and HJPC‐2. Briefly, removed tissue of approximately 1 × 1 cm^2^ was rinsed with phosphate buffered saline (PBS; OXOID Limited, Hampshire, England) containing 100 mg/ml P/S, cut into smaller pieces and added to 0.25% collagenase type II in EM without fetal calf serum and incubated at 37 °C for 3 h (Samee et al., 2008). After this time, the cells were centrifuged, the supernatant removed and the cells placed into a 25‐cm^2^ tissue‐culture flask in 2 ml of fresh EM. Fresh media was added to the flask every 2–3 days for 7 days. After 7 days, the culture media and nonadherent cells were removed (Chao et al., 2012; Dominici et al., 2006). hBMSCs were isolated from mononuclear cells (Lonza^®^, Castleford, UK). Briefly, mononuclear cells were treated with prewarmed basal culture media containing 0.1 mg/ml DNaseI (Stemcell™ technology, Grenoble, France). Cells were cultured at a density of 1.2 × 10^5^ cells/cm^2^ in 25‐cm^2^ tissue‐culture flasks. After 7 days, the non‐adherent cells were removed by washing and the adherent population termed passage 0.

### 2.4 Cell phenotyping

hBMSCs, hESMPs and HJPCs were detached from culture flasks using nonenzymatic cell disassociation solution, washed in PBS and resuspended in 1 ml of flow‐assisted cell sorting buffer (0.1% bovine serum albumin, 0.1% sodium azide, PBS 100 mm). Cell number was adjusted to 1 × 10^5^/ml and cells stained with anti‐CD90, anti‐CD105, anti‐CD146 and anti‐CD45 (human MSC multi‐colour flow cytometry kit, R&D system, Abingdon, UK) as per the manufacturer's instructions. Samples were analysed using a LSR II flow cytometry (BD Biosciences, Oxford, UK).

### 2.5 Application of fluid shear stress by rocking

For cell monolayers, HJPCs and hBMSCs were seeded in standard six‐well plates (Corning Inc., Amsterdam, The Netherlands) at a density of 10,000 cells/well in basal culture media. Cells were allowed to adhere for 24 h after media was replaced with osteogenic inducing media. For three‐dimensional (3D) cultures, hBMSC and hESMP were seeded onto the PCL scaffold (width 15 mm × length 35 mm × thickness 390 μm) and placed in the standard 6‐well plates with a sterile stainless steel ring on top to hold them in place. Scaffolds were immersed in 1 ml basal culture medium overnight before cell seeding; 10^5^ cells in 100 μl were seeded onto the scaffold and left for 24 h after which the cell seeded scaffolds were transferred to fresh plates and a coil of sterilized dental wire placed on top to submerge the scaffolds in the culture media. Medium was replaced with SM or OIM after a further 24 h.

For all conditions, samples were divided into a static condition (no‐flow) or subjected to rocking which would induce OFF (termed rocking group) using a standard see‐saw rocker (STR6 platform, Stuart Equipment, Stone, UK) starting from day 4 at 45 rpm for 1 h/day, for 5 days/week, at room temperature, based on previous published studies (Delaine‐Smith et al., 2012; Lim et al., 2013, Delaine‐Smith, 2013) . The static group were sham treated by placing on the laboratory bench for the same length of time but not subjected to rocking. Medium samples were collected on day 7, 14, or 21 for monolayer cultures, and day 28 for 3D cultures to evaluate VEGF). In each case the media were changed 48 h prior to the collection time point so that the media collected represented 48 h of cell secretion.

### 2.6 Resazurin reduction test for cell viability

The resazurin reduction test was used to measure cell viability as previously described (O'Brien, Wilson, Orton, & Pognan, 2000). Briefly, 0.1 mm resazurin solution salt solution in basal culture media was added to each sample and incubated for in the dark for 4 h at 37 °C; 200 μL of pink resorufin product was transferred to a microtitre plate, and measured spectrofluorometically (FLx800 Fluorescence Reader; BioTek, Potten, UK) at an excitation wavelength of 540/35 nm, and emission wavelength of 630/32 nm.

### 2.7 Total deoxyribose nucleic acid measurement and alkaline phosphatase activity

Total deoxyribose nucleic acid (DNA) was assessed using fluorescent QuantiT™ PicoGreen^®^ dsDNA reagent assay kit (Invitrogen, Paisley, UK) as per the manufacturer's instructions. Briefly, 500 μL of carbonate buffer was added to the cells for 30 min at 37 °C before scraping to remove cell lysate. Cell lysates were freeze‐thawed three times, vortexed and centrifuged. Cell lysate and PicoGreen^®^ solution were mixed and the fluorescence intensity measured spectrofluorometrically with an excitation of 480 nm and emission of 520 nm (Oliveira et al., 2006). The total DNA was calculated from the fluorescence emission to ng/ml using a standard curve. To determine alkaline phosphatase (ALP) activity, cell lysate was mixed with an Alkaline Phosphatase Yellow Liquid Substrate (Sigma–Aldrich) based on p‐nitrophenol phosphate and the absorbance measured using a spectrometer (ELx800; BioTeK, Potten, UK) at 405 nm every minute for 30 min. The enzyme activity was calculated as nmol of p‐nitrophenol/min (nmol pNP/min) and normalized to the total DNA from the same sample.

### 2.8 Collagen and calcium staining

Collagen and calcium staining was performed on days 21 and 28 for monolayer and 3D cultures, respectively. Total collagen production was measured by staining with 0.1% picrosirius red solution for 18 h. All unbounded dye was removed with distilled water (dH_2_O) and samples left to air‐dry. The samples were destained with sodium hydroxide (0.2 m) and methanol (ratio 1:1) with shaking at 50 rpm for 15 min. The absorbance of the resulting solution was measured in a spectrometer at 490 nm.

Total calcium deposition was measured by staining with 1 mg/ml alizarin red in dH_2_O, adjusted to pH 4.1 with ammonium hydroxide for 20 min at room temperature. All unstained dye was removed with dH_2_O and left to air‐dry. The stained samples were dissolved by adding 500 μl perchloric acid (5% *v*/v) for 30 min at room temperature and the absorbance of the eluted stain measured at 405 nm using a spectrometer.

### 2.9 μCT **imaging**

Mineralized matrix was also evaluated using μCT (Skyscan 1172, Kontich, Belgium). Samples were stacked and placed on a 20‐mm diameter brass tray. The μCT parameters were set at 17.5 μm/voxel resolution, two images for each point were merged, the filter was 1 mm of aluminium and a rotation step of 0.8°, 360° was applied. Approximately 750 slices were obtained. CTan and CTvol analysis software (Skyscan 1172, Kontich, Belgium) were used to quantify bone volume percentage (%BV) to evaluate mineral deposition on scaffolds and to reconstruct the 3D structure of the samples, respectively. The region of interest for measuring %BV, a diameter of 1.2 mm (inner diameter of metal ring) and height of 390 μm (approximate scaffold thickness) was selected and sectioned equally into top, mid and bottom portions (130 μm/region). Threshold values were calculated by analysing the greyscale index (GSI) distribution.

### 2.10 Assessment of vascular endothelial growth factor secretion

VEGF secretion into culture conditioned media was measured using a standard human VEGF ELISA Kit (PeproTech^®^, London, UK), following the manufacturer's instruction. Absorbance values were converted to VEGF concentration (pg/ml) using a standard curve and normalized to cell number determined from the resazurin reduction assay.

### 2.11 Statistics

Data are expressed as mean values ± standard error of the mean. Numbers of replicates are stated in the figure legend. *N* represents a biological repeat (separate experiment) and *n* represents a technical repeat (different samples within one experiment). Statistical analysis was performed using SPSS (IBM SPSS statistics 21). Cell viability, DNA quantification, ALP activity, calcium deposition, collagen production, and VEGF secretion were analysed using a Mann–Whitney *U* test. The differences were considered to be statistically significant at *p*≤0.05.

## 3 RESULTS

### 3.1 Rocking in combination with dexamethasone supplementation upregulates mineralization in monolayer cultures of primary adult hBMSCs and HJPCs

**Figure 1:**
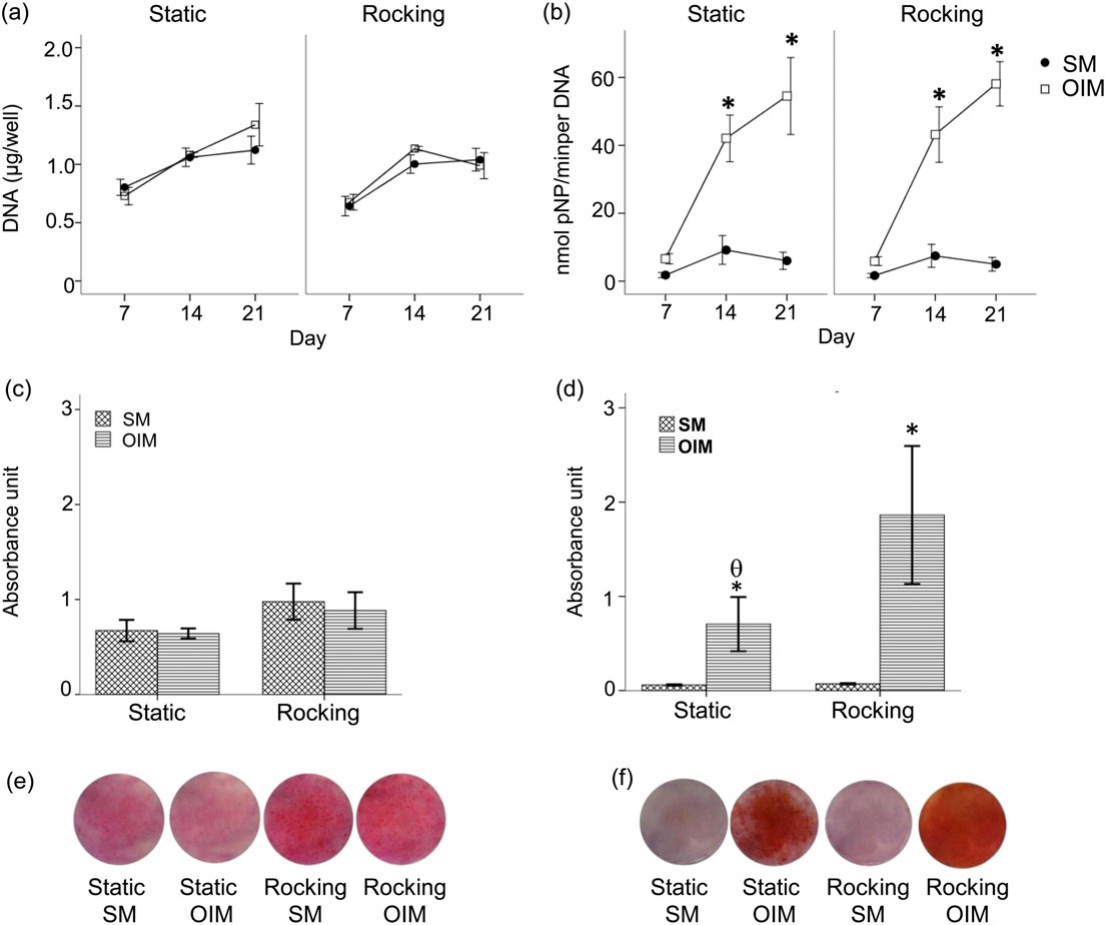
The effect of OFF on hBMSC matrix formation in monolayer culture in the presence (OIM) and absence of dexamethasone (SM) after 21 days. DNA (a), ALP activity normalized to DNA (μg/ml; b), collagen production by picrosirius red staining (c), and total calcium deposition by alizarin red staining (d). The photoimages show representative sets of picrosirius red staining (e) and alizarin red staining (f) on monolayer cultures of hBMSC. Data presented as mean ± standard error of the mean, (*N =* 3, *n =* 3), * = *p*<0.05 comparison between the SM and OIM groups, θ *= p*<0.05 comparison between the static and rocking group [Colour figure can be viewed at wileyonlinelibrary.com]

In monolayer culture (Figure 1a) the number of hBMSCs, as measured by total DNA, increased over time in all conditions, irrespective of the supplements added (SM vs. OIM) from day 7 to 21 while under rocking conditions cells in both media showed an increase to day 14 then no further increase by day 21.

The highest ALP activity was observed in the OIM conditions with no difference between the rocking and static groups (Figure 1b). In the absence of dexamethasone there was very little ALP production.

Collagen production was relatively low in all cells under all conditions (Figure 1c). Total calcium deposition however (Figure 1d) was stimulated in the OIM media group compared to the SM only group and further stimulated by rocking by day 21.

**Figure 2:**
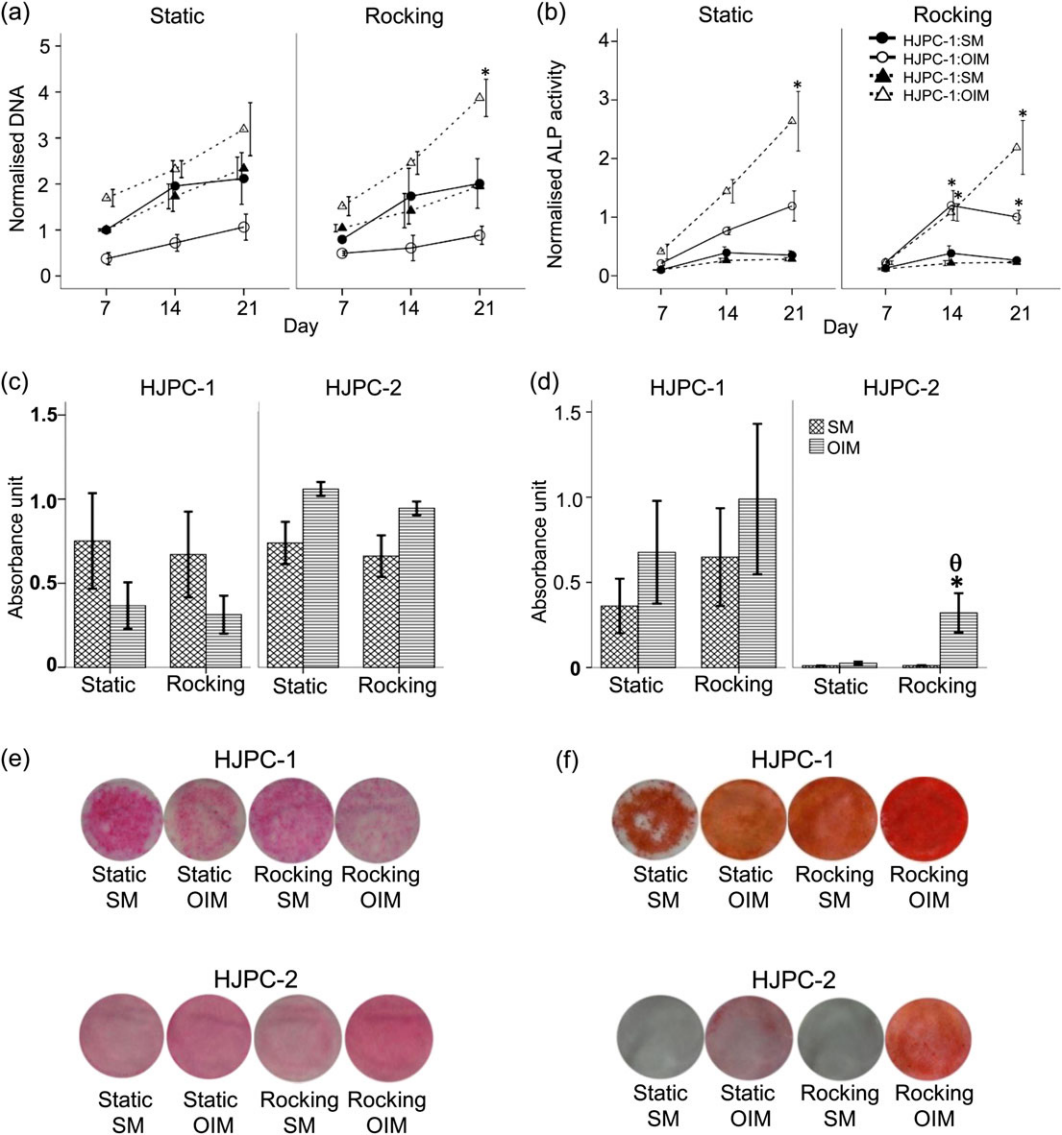
The effect of OFF on HJPC monolayer culture, for 28 (HJP‐1) days and 21 days (HJP‐2), in the absence (SM) or presence of Dex (OIM). Different time‐points are shown as the two groups of donor cells had different growth rates as seen in (a). Total DNA (a), ALP activity normalized to DNA (b), total collagen production by picrosirius red staining (c) , and total calcium deposition by alizarin red staining (d). The photoimages show representative set of picrosirius red (e) and alizarin red staining (f). Data is presented as mean ± SE, (*N =* 2, *n =* 3), * *= p*<0.05 comparison between the SM and OIM groups, θ *= p*<0.05 comparison between the rocking and static groups in the same medium [Colour figure can be viewed at wileyonlinelibrary.com]

HJPCs from two donors exhibited a fusiform mesenchymal stem cell‐like morphology similar to hBMSCs; however, hBMSCs appeared visibly larger than HJPC (Figure S2). The cell number, as measured by total DNA, of both HJPC‐1 and HJPC‐2 in monolayer culture increased under all conditions (SM and OIM groups) from 7 to 21 days (Figure 2a). The SM groups were similar to the OIM groups under both rocking and static conditions.

For HJPCs, ALP activity normalized to DNA (Figure 2b) was higher in the presence of dexamethasone for both donors at all time‐points. There was no significant difference between static and rocking conditions. For collagen production, the SM and OIM groups produced similar amounts of collagen in both static and rocking groups by day 21 (Figure 2c and e). However, total calcium deposition by the OIM rocking group was higher than for any other groups by day 21 (Figure 2d and f).

### 3.2 Rocking in combination with dexamethasone upregulates mineral deposition in cells cultured in electrospun scaffolds

**Figure 3:**
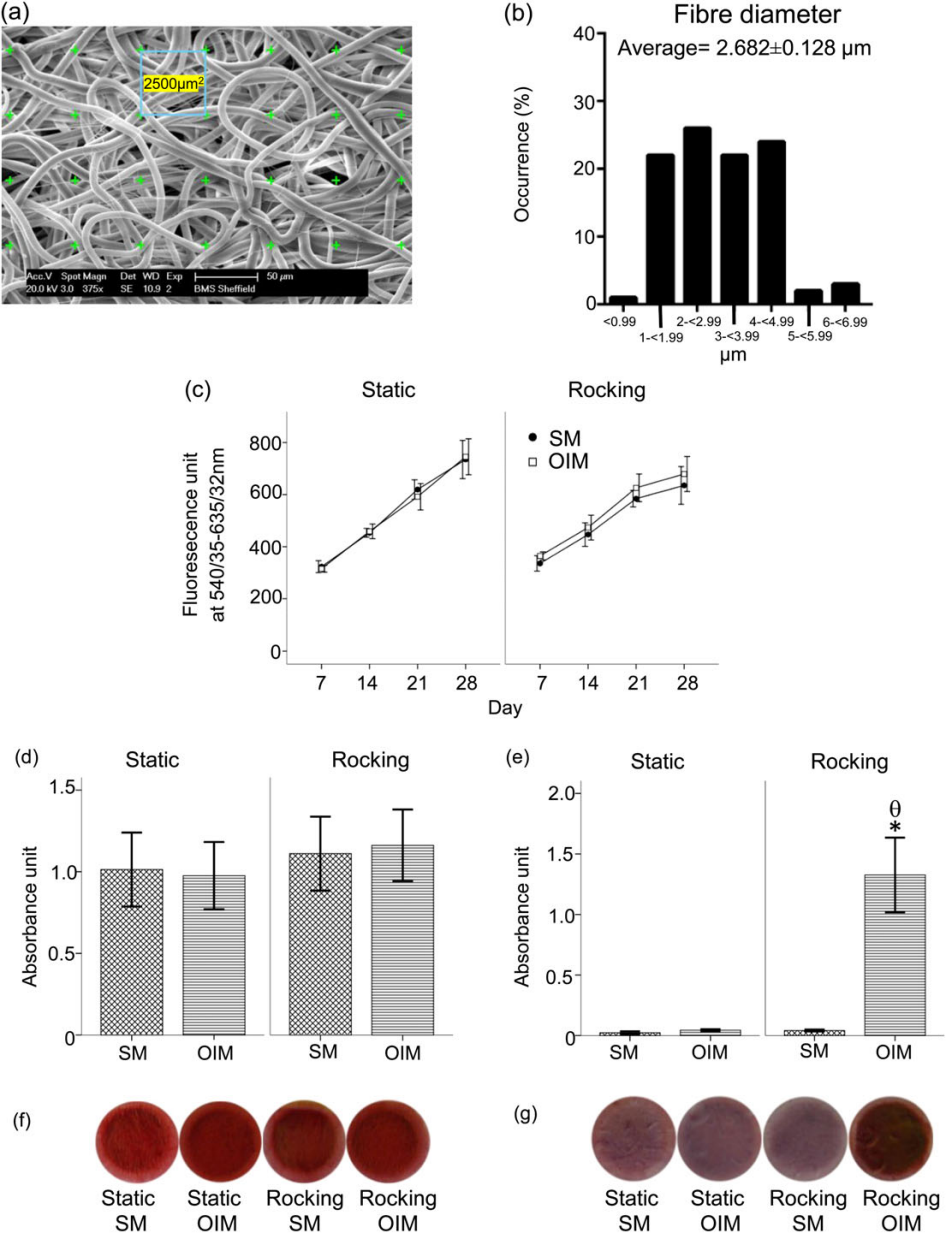
Top: (a) 10 wt% PCL electrospun scaffold characterisation and schematic of fibre diameter measurement from SEM images, (b) fibre diameter distribution and average fibre diameter of electrospun scaffolds. Scale bar 50 μm, mean ± standard error of mean (*n =* 100). Below: The effects of OFF on osteogenic differentiation of hBMSC cultured on 3D PCL electrospun scaffolds in the absence (SM) or presence of Dex (OIM). The viability of hBMSC was measured using a resazurin reduction test (c) for 28 days. Total collagen production was measured using picrosirius red staining (d) and total calcium deposition using alizarin red staining (e), after 28 days of culture. The photoimages show representative sets of picrosirius (f) and alizarin red (g) staining of hBMSC. Data presented as mean ± standard error of the mean, (*N =* 2, *n =* 3), * *= p*<0.05 comparison between the SM and OIM in the same condition, θ *= p*<0.05 comparison between the rocking and static groups in the same medium [Colour figure can be viewed at wileyonlinelibrary.com]

Electrospinning of PCL produced fibres with a smooth fibre morphology without bead formation. Representative SEM images of the 10wt% PCL randomised electrospun scaffolds spun using DCM as a solvent are shown in Figure 3a. The average fibre diameter of PCL was 2.7 μm (Figure 3b). In electrospun scaffolds, the cell viability of the hBMSCs increased in all conditions (SM and OIM) with no difference between these groups from day 7 to 28 (Figure 3c). There were no differences in collagen production between any conditions (Figure 3d, f). Total calcium deposition was greatest for cells cultured in OIM – this was significantly higher than for the other groups (*p*<0.05; Figure 3e, g).

**Figure 4:**
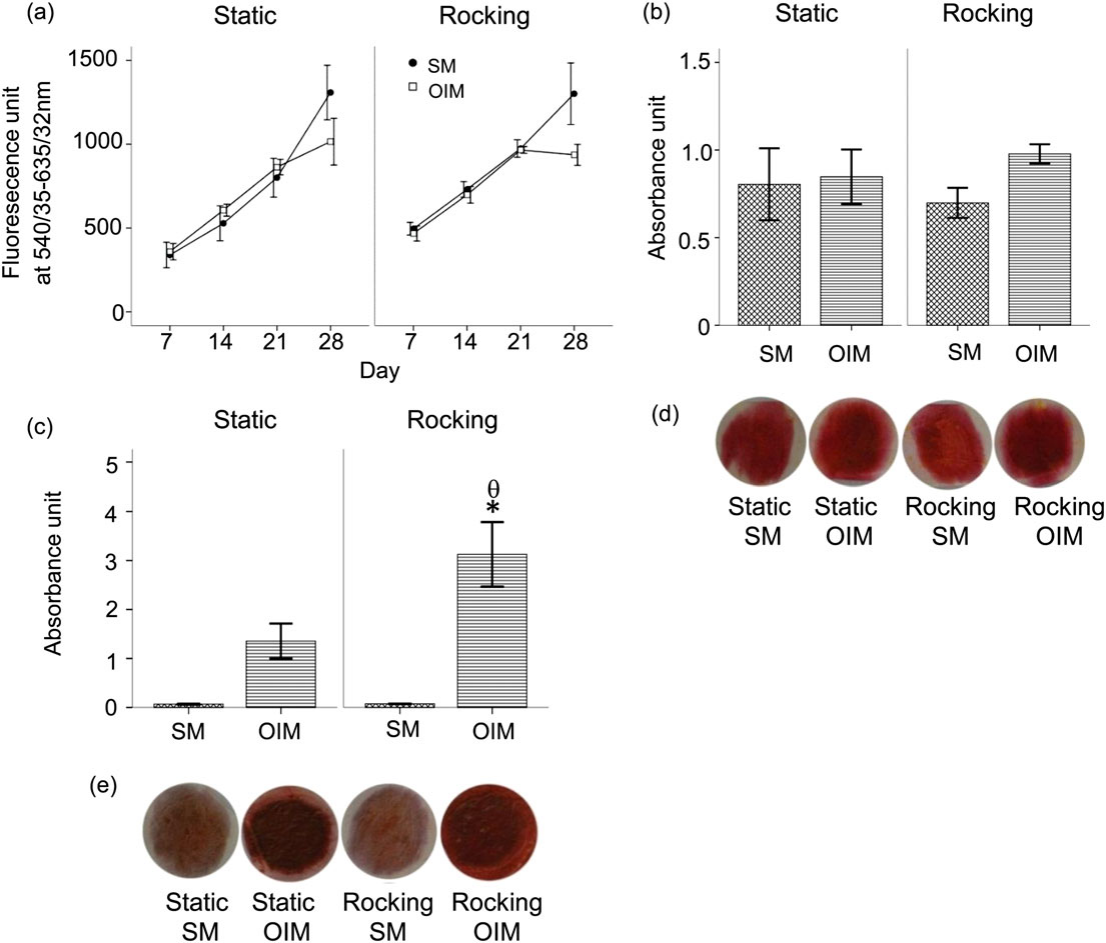
The effects of OFF on hESMPs cultured on PCL scaffolds in the absence (SM) or presence of Dex (OIM) for 28 days. The viability of hESMPs were measured using a reszasurin reduction test (a), total collagen production by picrosirius red staining (b) and total calcium deposition was measured by alizarin red staining (c). The photoimages show representative sets of picrosirius (d) and alizarin red (e) staining of hESMP. Data presented as mean ± standard error of the mean, (*N =* 2, *n =* 3), * = *p*<0.05 comparison between SM and OIM, θ = *p*<0.05 comparison between the rocking and static conditions [Colour figure can be viewed at wileyonlinelibrary.com]

The cell viability of hESMPs (mesenchymal progenitors) seeded in electrospun scaffolds increased over time in all conditions from day 7 to 28 (Figure 4a). There were no differences in collagen production between cells cultured under any conditions (Figure 4b and d). Total calcium deposition by the rocking OIM group was significantly higher compared to the other groups (*p*<0.05; Figure 4c and e). The SM groups deposited negligible calcium compared to the OIM groups irrespective of whether they were rocked or not.

**Figure 5:**
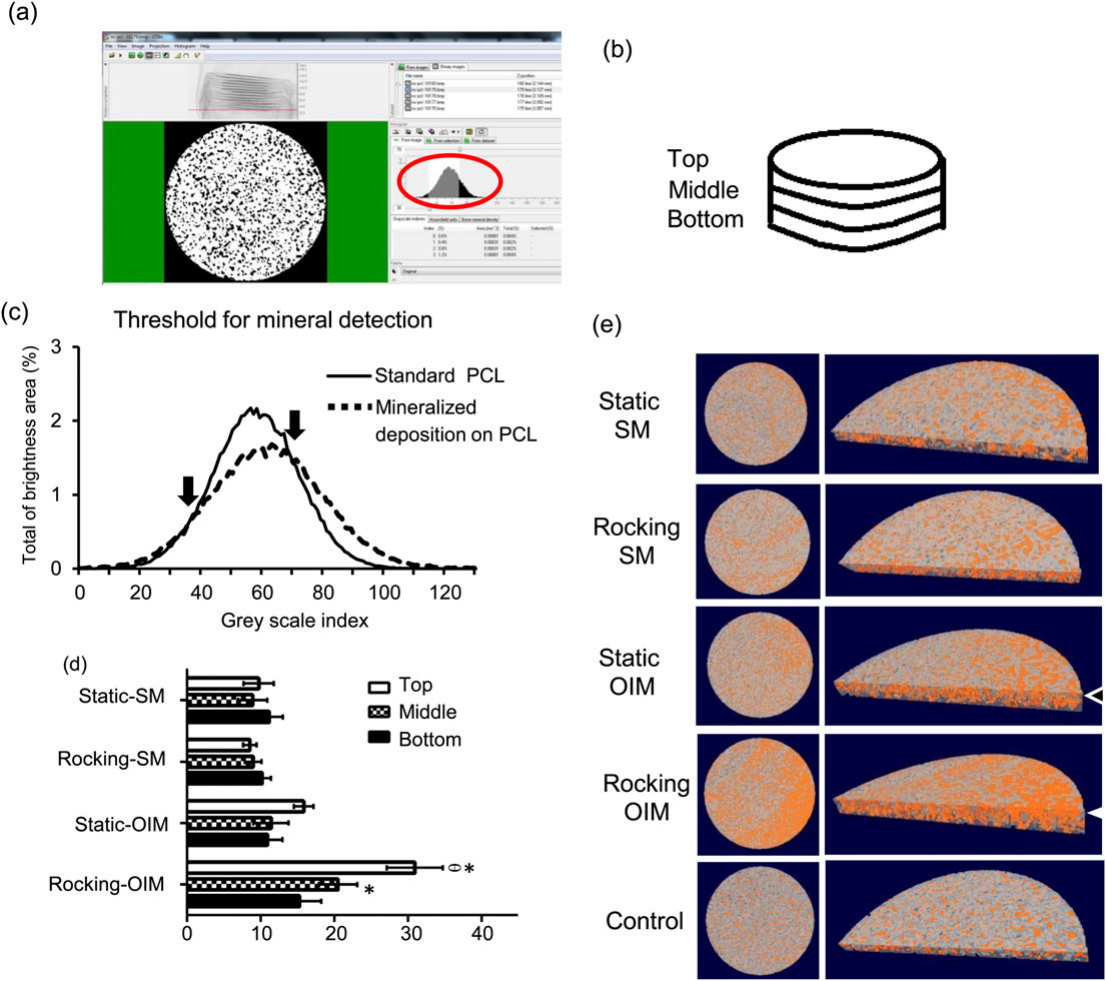
Top: (a) A window from CTanalyze software presenting the greyscale distribution histogram (red circle). (b) Schematic of the section of the electrospun scaffold that was used to analyse calcium deposition (bone volume percentage). The cylindrical region of interest, diameter 1.2 mm. The scaffold was divided into three layers with 130 μm in each layer: top, middle, and bottom. The greyscale distribution histogram of standard PCL and calcium deposition on the scaffolds was used for setting the thresholds (c). The range between 30 and 70 GSI served to identify standard PCL scaffolds. The range between 70 and 125 GSI was determined to identify calcium deposition (*N =* 1, *n =* 3). Below: The effects of OFF on hESMP calcium deposition cultured on PCL scaffolds in the absence (SM) or presence of Dex (OIM) for 28 days. The top, middle, and bottom of percentile bone volume (%BV) with subtraction of standard PCL scaffolds measured using CTanalyze (d). Data presented as mean ± standard error of mean, (*N =* 2, *n =* 3), * = *p*<0.05 comparison between SM and OIM. θ = *p*<0.05 comparison between the rocking and static conditions in the same level and medium. μCT images of calcium deposition (orange area) in the scaffolds within the cylindrical region of interest (e). The circle images show the top surface of the scaffolds and half circle images showed the cross‐section of scaffolds. The top level of the rocking‐OIM group contained more calcium than the static‐OIM group (white arrow heads) [Colour figure can be viewed at wileyonlinelibrary.com]

Mineralization was also studied using μCT analysis as described in Figure 5. Figure 5a describes the approach in which scaffolds were divided into 3 layers (Figure 5b) and examined using a GSI threshold for mineral detection. GSI in the range of 30–70 was found for PCL scaffolds without cells, while PCL scaffolds plus cells (hESMPs) cultured in OIM with rocking produced calcium deposits that could be detected with a GSI of ≥70 as shown in Figure 5c. This group was chosen for thresholding as calcium deposition was demonstrated in these constructs based on alizarin red staining (Figure 4c). The GSI values for the scaffolds without any cells were subtracted from GSI values for scaffolds plus cells to determine mineral deposition in the samples.

It was evident that mineralized matrix was not evenly distributed throughout the scaffolds. A side view image of a transverse section throughout the scaffold from the rocking OIM group shows that the top region contains more mineral than the other layers (white arrowhead) and the top layer of the static OIM group (black arrowhead; Figure 5e). For the rocking OIM group there was significantly higher matrix in the upper region than in the middle and bottom regions (*p*<0.05; Figure 5d). The middle region of the rocking OIM group contains more mineral than the middle region of the static OIM group.

### 3.3 Effect of dexamethasone and rocking on VEGF secretion

**Figure 6:**
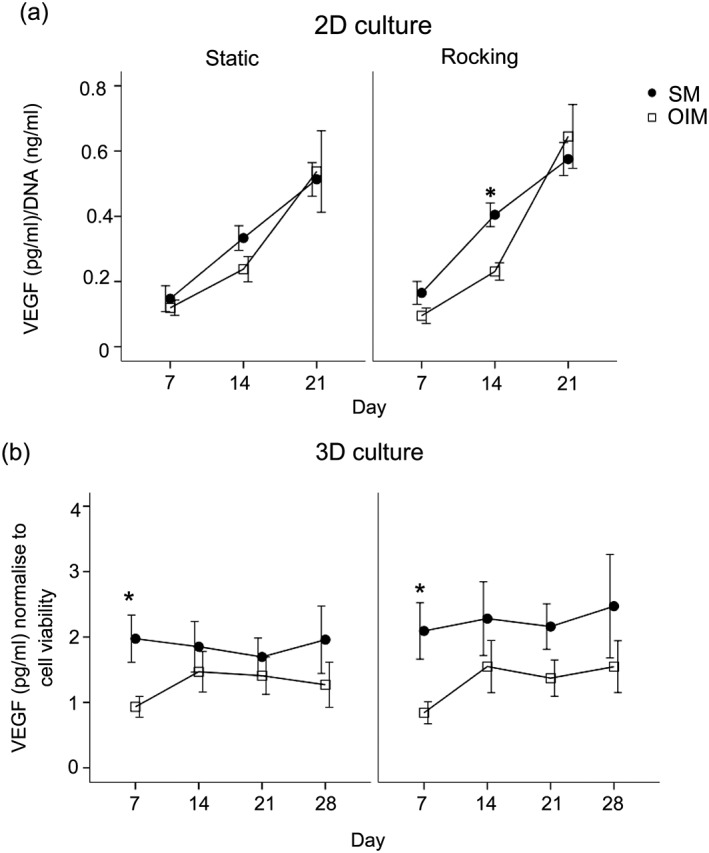
The effects of OFF of VEGF secretion in hBMSC cultured as monolayers or 2D culture (a) or in PCL electrospun scaffolds or 3D culture (b) in the absence (SM) or presence of Dex (OIM). VEGF secretion over 28 days (during a 48‐h collection period) normalized to viable cell number by resazurin assay. Data presented as mean ± standard error of the mean, (*N =* 2, *n =* 3), * *= p*<0.05 comparison between the SM and OIM in the same condition

The secretion of VEGF (sampled by collecting media over a 48‐h collection period) by hBMSC monolayers increased overtime under all conditions (Figure 6a). There was no significant effect of media or of rocking on VEGF secretion for these cells.

For cells cultured in 3D scaffolds VEGF secretion was detected from day 7 to day 28 at similar concentrations. VEGF levels in the absence of dexamethasone were slightly greater but this was not statistically significant. There was no effect of rocking on VEGF secretion (Figure 6b).

## 4 DISCUSSION

This study has shown that primary bone marrow and jaw periosteal osteoprogenitor cells (HBMSCs and HJPCs) respond to rocking induced OFF as a mechanical stimulus with an increase in mineralization, as predicted by previous data on the embryonic cell line hESMPs. Subsequently, hBMSCs and hESMPs, as cell sources that yield high numbers, were used to assess whether this affect could be used to stimulate the cells while in the 3D environment of an electrospun scaffold and showed that indeed mineralization in 3D can be stimulated by rocker culture.

Bone tissue engineering aims to provide a biocompatible material capable of substituting for autologous bone grafts for surgical reconstruction. The challenge for cleft palate repair is to fabricate scaffolds for the hard part of the palate to allow normal facial development. Further requirements are that the polymer scaffold is biodegradable and has the appropriate mechanical strength and biological properties to behave as a temporary extracellular matrix (ECM) until the supported cells are able to synthesize their own ECM to enable regeneration. In order to do this electrospun PCL was chosen as the test material because of its biocompatibility, high porosity, slow degradation rate, and ability to support bone forming cells. For example an *in vivo* study found that PCL scaffolds degraded by about 39 ± 1% after 28 days of implantation in mice more slowly than polylactic‐glycolic acid copolymer (50:50) (Sung, Meredith, Johnson, & Galis, 2004). Moreover, PCL electrospun scaffolds partially mimic the fibrous architecture of collagenous ECM and support good cell adhesion, proliferation, and osteogenic differentiation (Hutmacher et al., 2001).

All cell types used in this study were analysed for their surface antigen expression. Both hESMP and hBMSC were confirmed to be MSCs by expression of CD146, CD105, and CD90 and the lack of expression of CD45 (Table S3), which are key makers for MSCs (Tormin et al., 2011). CD45 would indicate the presence of haematopoietic cells which may contaminate the osteoprogenitor cells. However, there was no evidence of CD146 in the HJPC group. With the caveat that these data were obtained from passaged cells and should be verified in freshly isolated cells, it is suggested that the lack of CD146 relates to the cell's origins. CD146 is known as a melanoma cell adhesion molecule and it has been found to be present in human bone marrow cells that contribute to the vascular niche but not on cells that contribute to the osteoblastic niche (Sloan & Waddington, 2009). This may be expected as the periosteum is a membrane beneath which bone formation occurs whereas hBMSC are derived from bone marrow, which also contains a vascular niche.

OFF can enhance osteogenic differentiation and therefore may be of use as a pretreatment in cleft palate bone tissue engineering. It was previously shown that OFF applied using similar conditions slightly upregulates ALP activity and collagen production and strongly upregulates calcium production in monolayer culture by hESMPs (Delaine‐Smith et al., 2012). Others also applied OFF to monolayer cultures of hBMSC or human fetal osteoblastic cells using a parallel plate flow chamber at a peak shear stresses of up to 2 Pa (Li et al., 2004). Fluid flow under an orbital shaker has also been shown to enhance chondrogenesis in HJPC (Ferretti & Mattioli‐Belmonte, 2014, Tarng et al., 2010). To the authors' knowledge this is the first study to stimulate the osteogenesis of HJPC using fluid flow and here it is demonstrated that this stimulus enhances mineralized matrix production in a similar way as to that previously demonstrated for other osteogenic progenitors. HJPC are derived from the patient jaw periosteum tissue which is easily removed from the donor site while the patient is undergoing palatal closure surgery. By contrast, hBMSCs were collected from another harvesting region (hip bone); therefore, patients may suffer from donor site morbidity and more time consuming surgical treatment to source these cells. While these data indicate some promising characteristics of HJPs there were fewer cells than would be obtained from a typical bone marrow sample and so it will be important to understand whether faster proliferating cells or larger tissue samples can be obtained from younger donors.

There were some small but important differences between the previously demonstrated response of hESMP cells to the rocking stimulus and that presented here for primary cells. For example: here there was no effect of OFF on ALP activity or collagen production whereas these were reported to be slightly higher after rocking in hES‐MP cells (Delaine‐Smith et al., 2012). However, it has been well demonstrated that there is high donor variability in the ALP response of individual human donors to an osteogenic stimulus (Osyczka, Damek‐Poprawa, Wojtowicz, & Akintoye, 2009; Sittichokechaiwut, Edwards, Scutt, & Reilly, 2010), as can be also seen by the error bars in Figures 1b and 2b, and that ALP peaks during osteogenic differentiation (Lian & Stein, 1995), and therefore the apparent ALP response is highly dependent on the analysis time‐points. Here, ALP did not appear to have reached a peak before 21 days. This result is consistent with the study of Nauman, Satcher, Keaveny, Halloran, and Bikle (2001) who found no effect of fluid flow on ALP activity measured on day 14.

Collagen deposition can be stimulated by a mechanical stimulus under certain conditions for example BMSCs cultured on 3D scaffolds and stimulated in a bioreactor (Sikavitsas, Bancroft, Holtorf, Jansen, & Mikos, 2003; Sittichokechaiwut et al., 2010; Zhou et al., 2011). However, in this study no effect of OFF was demonstrated in the cell types examined. This result is similar to the study by Li et al. (2004) who subjected MSCs to OFF in a parallel plate flow chamber and found the gene expression of collagen type I was not affected. Filipowska, Reilly, and Osyczka (2016) found that perfusion flow of hBMSC in porous polyurethane scaffolds could enhance osteogenic potential but had no effect on collagen production.

This study found that calcium deposition, indicative of bone‐like matrix, in the rocking groups under the OIM medium in both hBMSC and HJPC was higher than in the SM medium. The combination of OFF and Dex appears to further enhance osteoblastic differentiation especially at the late stages. Interestingly, when multiple modes of mechanical stimulation of MSCs are compared, it is consistently found that calcium deposition is the most strongly affected by the mechanical stimulus (Delaine‐Smith et al., 2012; Sittichokechaiwut et al., 2010).

Having established the suitability of rocker culture to stimulate mineralization in monolayer in cell lines and primary cells it was tested whether this culture method could be applied to 3D scaffolds. To the best of the authors' knowledge, no study has used a standard see‐saw rocker as a simple method for mechanically stimulating cells with OFF for cell culture on electrospun scaffolds. Here, it was demonstrated that subjecting cell‐seeded electrospun PCL scaffolds to short bouts of OFF on a rocker in the presence of Dex caused greater calcium deposition compared to static cultures. The effect was very similar in both embryonic derived hESMP, used here as a model cell line, due to their capacity to reliably mineralize in 3D culture, and primary adult hBMSC which are well‐known osteoprogenitor cells suitable for clinical study. The results presented here suggest that the combination of OFF and Dex during culture synergistically stimulate the osteogenic differentiation of mesenchymal progenitors on PCL scaffolds. Although there were not sufficient mesenchymal progenitors available from the periosteal source for 3D culture of HJPCs it is predicted that HJPCs could also be induced to mineralize in electrospun scaffolds by this method.

Fluid flow has been shown to enhance osteogenic differentiation of osteoprogenitor cells and calcium deposition by several pathways such as the stretch‐activated ion channels, gap junctions, focal adhesion complexes, paracrine signalling such as prostaglandin E2 (PGE_2_) and the primary cilia (Delaine‐Smith, Sittichokechaiwut, et al., 2014; Janmey & McCulloch, 2007). Lastly, OFF can stimulate the mineralization of cells by enhanced nutrient distribution and oxygen transportation to cells in scaffolds compared to static culture (McCoy & O'Brien, 2010). The electrospun PCL scaffolds in this study are thin (300‐μm thickness) compared to typical porous scaffolds subjected to bioreactor culture therefore nutrient diffusion should be less of a limitation to static culture compared to other studies. However, it has been suggested that cells should not be >200 μm from the nutrient source therefore cells in the centre of the electrospun scaffolds may still have experienced limited diffusion, especially as matrix forms and fills up the pores in longer term static cultures (Muschler, Nakamoto, & Griffith, 2004).

For hBMSC the effect of rocking in electrospun scaffolds was very similar to in monolayer, with the rocking group under Dex supplemented medium having the highest calcium deposition. It is interesting that the effect of Dex in the 3D culture is very small and not statistically significant (Figure 3e) compared to in 2D culture (Figure 1d). And therefore the difference that rocking makes in 3D is much greater, leading to 10‐fold more calcium deposition compared to Dex alone. Kale et al. (2000) and Yamaguchi, Ohno, Sato, Kido, and Fukushima (2014) reported that bone cells in 3D culture deposited more calcium and noncollagenous protein such as ALP and osteonectin than in monolayer culture. This might be because the higher density of bone cells facilitates greater cell–cell communication and interaction compared with 2D culture.

μCT has advantages as a tool for real‐time monitoring for tissue engineering as it is a nondestructive imaging technique used to evaluate quantitative mineralization in the scaffolds (Cartmell, Huynh, Lin, Nagaraja, & Guldberg, 2004; Hagenmueller et al., 2007; Thimm, Wechsler, Bohner, Muller, & Hofmann, 2013). In addition, it has been used widely in biomaterials for analysis of animal models because there is no need to biopsy the bone tissue for evaluation. The characterization of mineral deposition on electrospun scaffolds using μCT has not been reported in many studies. The threshold for quantitative μCT analysis of bone usually used is the global threshold or automate global threshold such as Otsu's method (1979). However, this method has disadvantages as small amount of mineralized deposition would not be detected (Parkinson, Badiei, & Fazzalari, 2008). Individual threshold determinations were used in this study. As demonstrated by alizarin red staining the combination of both Dex and rocking produced significantly higher mineral deposition on the electrospun scaffolds. However, Dex alone did not increase the amount of mineral visualized by the μCT technique and the differences in mineralization where much smaller than those detected by alizarin red staining. Therefore, it appears that the μCT was not able to detect small mineral deposits that were detectable by alizarin red. Interestingly the ability of μCT to localize the deposited mineral demonstrated that there was a higher percentage of mineral deposition at the top level of the rocked scaffolds than the middle and bottom sections (Figure 5d). The reasons for this were probably because in the rocker the fluid would have flowed over the scaffold surface and the flow magnitude would gradually decrease with scaffold depth assuming the scaffold is firmly attached to the well base (scaffolds remained in the same orientation with respect to the well base throughout the culture period). A more even distribution of mineral may be obtained by turning the scaffold over during rocking or by adapting the culture system such that the scaffold is suspended in flowing fluid. One would need a computational model of fluid flow through a porous medium to calculate what the shear stress is on the walls of the electrospun fibres, such as those used for other types of porous scaffolds (Birmingham, Grogan, Niebur, Mcnamara, & McHugh, 2013; Marin & Lacroix, 2015).

Vascularization is an important process during bone repair initiated by recruiting endothelial cells for blood vessel formation to provide nutrients and excrete waste from the regenerating bony matrix. In this study cells secreted VEGF in all conditions and although VEGF was slightly inhibited by Dex this recovered at later time‐points. Work has also indicated that Dex may inhibit VEGF (Heiss et al., 1996). The rising levels of VEGF indicate that if implanted, the cells in the scaffolds would support recruitment of endothelial cells. However, there were no differences in VEGF secretion by hBMSC between the rocking and static groups in either monolayer or 3D culture. This did not support the hypothesis that VEGF would be upregulated by rocker culture. VEGF secretion has been shown to be markedly stimulated by pulsatile fluid flow (Thi, Suadicani, & Spray, 2010; Yuan, Sakamoto, Song, & Sato, 2013), in non Dex‐containing media. However, in another study, human MSCs subjected to OFF in rocker culture showed only a small (19%) increase in VEGF secretion. This indicates there is a complex relationship between the flow stimulus, Dex and VEGF secretion. In general, VEGF has been shown to be upregulated in parallel to mineralization, the rocker regimen used in this study, which strongly supported mineral deposition, did not appear to be correlated with higher VEGF. However, VEGF was measured in the media as a product of all the cell's activity and the μCT scan results indicate that mineralization was localized more strongly to the upper surface; therefore, it would be interesting to observe whether there were regional differences in VEGF secretion throughout the scaffolds.

## 5 CONCLUSIONS

This study indicated that a rocking stimulus previously described as supporting mineralization of embryonic‐derived progenitors in monolayer can be applied to primary more clinically relevant osteoprogenitor cells in both monolayer and 3D to stimulate mineralization. Furthermore, it is confirmed that electrospun PCL is a suitable scaffold material for bone tissue engineering as it showed good biocompatibility, cell attachment and allowed osteogenic differentiation of human osteogenic progenitor cells. HJPC responded to OIM and rocker stimulation in a similar manner to hBMSC, suggesting that these cells have potential as autologous osteogenic progenitor cells. HJPC might be a new cell source for cleft palate repair, which could be collected from palate closure surgery, at a patient age of about 1 year. The introduction of OFF induced by a standard see‐saw rocker further increased the mineralization of cells in Dex supplemented culture conditions in both monolayer and 3D scaffolds, particularly on the upper surface of the scaffolds. This would be a cost‐effective and easy to scale‐up method for prestimulation of tissue engineered constructs. It is concluded that an oscillatory flow stimulus could stimulate hard tissue formation from human osteogenic progenitor cells on thin tissue engineered constructs for cleft palate repair and other oral maxillofacial reconstructions such as alveolar ridge augmentation for tooth implantation and guided bone tissue regeneration for periodontal surgery.

## SPONSOR

Naresuan University Scholarship to SP and EPSRC scholarship to RMDS.

## Supporting information

The supporting information indicates the the cell morphology of hBMSCs and HJPCs from two donors, and surface antigen expression patterns by FACS analysis of hESMPs and HJPCs from two donors.

**FIGURE S1.** Schematic diagram detailing cell types, experimental set‐up and end‐point assays used in this study.

**FIGURE S2.** Phase contrast images of hBMSC and HJPC from two donors on day 7 of culture in SM medium. The cell morphology of hBMSC (a) is a fusiform mesenchymal cell0like morphology. HJPC‐1 (b) and HJPC‐2 (c) both have fusiform mesenchymal stem cell‐like morphologies (white arrows) but hBMSCs were larger the HJPCs. Scale bar = 200 μm.

**Table S1:** Surface antigen expression patterns of hESMP, HJPC‐1, HJPC‐2, hBMSC‐1, hBMSC‐2, and hBMSC‐3were measured using flow‐assisted cell sorting.

## References

[term2462-bib-0001] Behnia, H. , Khojasteh, A. , Soleimani, M. , Tehranchi, A. , & Atashi, A. (2012). Repair of alveolar cleft defect with mesenchymal stem cells and platelet derived growth factors: A preliminary report. Journal of Cranio‐Maxillofacial Surgery, 40(1), 2–7. 21420310 10.1016/j.jcms.2011.02.003

[term2462-bib-0002] Birmingham, E. , Grogan, J. A. , Niebur, G. L. , McNamara, L. M. , & McHugh, P. E. (2013). Computational modelling of the mechanics of trabecular bone and marrow using fluid structure interaction techniques. Annals of Biomedical Engineering, 41(4), 814–826. 23519534 10.1007/s10439-012-0714-1

[term2462-bib-0003] Bye, F. J. , Bissoli, J. , Black, L. , Bullock, A. J. , Puwanun, S. , Moharamzadeh, K. , … MacNeil, S. (2013). Development of bilayer and trilayer nanofibrous/microfibrous scaffolds for regenerative medicine. Biomaterials Science, 1, 942–951. 10.1039/c3bm60074b 32481963

[term2462-bib-0004] Cartmell, S. , Huynh, K. , Lin, A. , Nagaraja, S. , & Guldberg, R. (2004). Quantitative microcomputed tomography analysis of mineralization within three‐dimensional scaffolds in vitro. *Journal of Biomedical Materials Research* . Part A, 69A(1), 97–104. 10.1002/jbm.a.20118 14999756

[term2462-bib-0005] Chao, Y. H. , Wu, H. P. , Chan, C. K. , Tsai, C. , Peng, C. T. , & Wu, K. H. (2012). Umbilical cord‐derived mesenchymal stem cells for hematopoietic stem cell transplantation. Journal of Biomedicine & Biotechnology, 2012, 759503. 23093863 10.1155/2012/759503 PMC3471031

[term2462-bib-0006] De Bari, C. , Dell'accio, F. , Vanlauwe, J. , Eyckmans, J. , Khan, Y. M. , Archer, C. W. , … Luyten, F. P. (2006). Mesenchymal multipotency of adult human periosteal cells demonstrated by single‐cell lineage analysis. Arthritis and Rheumatism, 54(4), 1209–1221. 16575900 10.1002/art.21753

[term2462-bib-0008] Delaine‐Smith, R. (2013). Mechanical and physical guidance of osteogenic differentiation and matrix production *.* Doctor of Philosophy, The University of Sheffield.

[term2462-bib-0009] Delaine‐Smith, R. M. , MacNeil, S. , & Reilly, G. C. (2012). Matrix production and collagen structure are enhance in two types of osteogenic progenictor cells by a simple fluid shear stress stimulus. European Cells & Materials, 24, 162–174. 22865228 10.22203/ecm.v024a12

[term2462-bib-0010] Delaine‐Smith, R. M. , Green, N. H. , Matcher, S. J. , MacNeil, S. , & Reilly, G. C. (2014). Monitoring fibrous scaffold guidance of three‐dimensional collagen organisation using minimally‐invasive second harmonic generation. PLoS One, 9(2). e89761. 10.1371/journal.pone.0089761 PMC3938545 24587017

[term2462-bib-0011] Delaine‐Smith, R. M. , Sittichokechaiwut, A. , & Reilly, G. C. (2014). Primary cilia respond to fluid shear stress and mediate flow‐induced calcium deposition in osteoblasts. FASEB Journal, 28(1), 430–439. 24097311 10.1096/fj.13-231894 PMC4012163

[term2462-bib-0012] Dominici, M. , Le Blanc, K. , Mueller, I. , Slaper‐Cortenbach, I. , Marini, F. C. , Krause, D. S. , … Horwitz, E. M. (2006). Minimal criteria for defining multipotent mesenchymal stromal cells. The International Society for Cellular Therapy position statement. Cytotherapy, 8(4), 315–317. 16923606 10.1080/14653240600855905

[term2462-bib-0013] Ferretti, C. , & Mattioli‐Belmonte, M. (2014). Periosteum derived stem cells for regenerative medicine proposals: Boosting current knowledge. World Journal of Stem Cells, 6(3), 266–277. 25126377 10.4252/wjsc.v6.i3.266 PMC4131269

[term2462-bib-0014] Filipowska, J. , Reilly, G. , & Osyczka, A. (2016). A single short session of media perfusion induces osteogenesis in hBMSCs cultured in porous scaffolds, dependent on cell differentiation stage. Biotechnology and Bioengineering, 113(8), 1814–1824. 26806539 10.1002/bit.25937

[term2462-bib-0015] Gurkan, U. A. , & Akkus, O. (2008). The mechanical environment of bone marrow: A review. Annals of Biomedical Engineering, 36(12), 1978–1991. 18855142 10.1007/s10439-008-9577-x

[term2462-bib-0016] Hagenmueller, H. , Hofmann, S. , Kohler, T. , Merkle, H. P. , Kaplan, D. L. , Vunjak‐Novakovic, G. , … Meinel, L. (2007). Non‐invasive time‐lapsed monitoring and quantification of engineered bone‐like tissue. Annals of Biomedical Engineering, 35(10), 1657–1667. 17546503 10.1007/s10439-007-9338-2

[term2462-bib-0017] Heiss, J. D. , Papavassiliou, E. , Merrill, M. J. , Nieman, L. , Knightly, J. J. , Walbridge, S. , … Oldfield, E. H. (1996). Mechanism of dexamethasone suppression of brain tumor‐associated vascular permeability in rats ‐ Involvement of the glucocorticoid receptor and vascular permeability factor. Journal of Clinical Investigation, 98(6), 1400–1408. 8823305 10.1172/JCI118927 PMC507566

[term2462-bib-0018] Hibi, H. , Yamada, Y. , Ueda, M. , & Endo, Y. (2006). Alveolar cleft osteoplasty using tissue‐engineered osteogenic material. International Journal of Oral and Maxillofacial Surgery, 35(6), 551–555. 16584868 10.1016/j.ijom.2005.12.007

[term2462-bib-0020] Hutmacher, D. W. , Schantz, T. , Zein, I. , Ng, K. W. , Teoh, S. H. , & Tan, K. C. (2001). Mechanical properties and cell cultural response of polycaprolactone scaffolds designed and fabricated via fused deposition modeling. Journal of Biomedical Materials Research, 55(2), 203–216. 11255172 10.1002/1097-4636(200105)55:2<203::aid-jbm1007>3.0.co;2-7

[term2462-bib-0021] Janmey, P. A. , & McCulloch, C. A. (2007). Cell mechanics: Integrating cell responses to mechanical stimuli. Annual Review of Biomedical Engineering, 9, 1–34. 10.1146/annurev.bioeng.9.060906.151927 17461730

[term2462-bib-0022] Jha, B. S. , Ayres, C. E. , Bowman, J. R. , Telemeco, T. A. , Sell, S. A. , Bowlin, G. L. , & Simpson, D. G. (2011). Electrospun collagen: a tissue engineering scaffold with unique functional properties in a wide variety of applications. Journal of Nanomaterials, 2011, 1–15.21808638

[term2462-bib-0023] Kale, S. , Biermann, S. , Edwards, C. , Tarnowski, C. , Morris, M. , & Long, M. W. (2000). Three‐dimensional cellular development is essential for *ex vivo* formation of human bone. Nature Biotechnology, 18(9), 954–958. 10.1038/79439 10973215

[term2462-bib-0024] Khojasteh, A. , Eslaminejad, M. B. , & Nazarian, H. (2008). Mesenchymal stem cells enhance bone regeneration in rat calvarial critical size defects more than platelete‐rich plasma. Oral Surgery Oral Medicine Oral Pathology Oral Radiology and Endodontology, 106(3), 356–362. 10.1016/j.tripleo.2007.10.017 18424120

[term2462-bib-0026] Li, Y. J. , Batra, N. N. , You, L. D. , Meier, S. C. , Coe, I. A. , Yellowley, C. E. , & Jacobs, C. R. (2004). Oscillatory fluid flow affects human marrow stromal cell proliferation and differentiation. Journal of Orthopaedic Research, 22(6), 1283–1289. 15475210 10.1016/j.orthres.2004.04.002

[term2462-bib-0027] Lian, J. B. , & Stein, G. S. (1995). Development of the osteoblast phenotype: molecular mechanisms mediating osteoblast growth and differentiation. The Iowa Orthopaedic Journal, 15, 118–140. 7634023 PMC2329080

[term2462-bib-0028] Lim, K. T. , Kim, J. , Seonwoo, H. , Chang, J. U. , Choi, H. , Hexiu, J. , … Chung, J. H. (2013). Enhanced osteogenesis of human alveolar bone‐derived mesenchymal stem cells for tooth tissue engineering using fluid shear stress in a rocking culture method. *Tissue Engineering* . Part C‐Methods, 19(2), 128–145. 10.1089/ten.tec.2012.0017 23088630

[term2462-bib-0029] Lohberger, B. , Payer, M. , Rinner, B. , Kaltenegger, H. , Wolf, E. , Schallmoser, K. , … Jakse, N. (2013). Tr‐lineage potential of intraoral tissue‐derived mesenchymal stromal cells. Journal of Cranio‐Maxillofacial Surgery, 41(2), 110–118. 22898339 10.1016/j.jcms.2012.06.001

[term2462-bib-0030] Marin, A. C. , & Lacroix, D. (2015). The inter‐sample structural variability of regular tissue‐engineered scaffolds significantly affects the micromechanical local cell environment. Interface Focus, 5(2). 10.1098/rsfs.2014.0097 PMC4342953 25844157

[term2462-bib-0031] McCoy, R. J. , & O'Brien, F. J. (2010). Influence of shear stress in perfusion bioreactor cultures for the development of three‐dimensional bone tissue constructs: A review. Tissue Engineering. Part B, Reviews, 16(6), 587–601. 20799909 10.1089/ten.TEB.2010.0370

[term2462-bib-0032] Mossey, P. A. , Little, J. , Munger, R. G. , Dixon, M. J. , & Shaw, W. C. (2009). Cleft lip and palate. Lancet, 374(9703), 1773–1785. 19747722 10.1016/S0140-6736(09)60695-4

[term2462-bib-0033] Moy, P. K. , Lundgren, S. , & Holmes, R. E. (1993). Maxillary sinus augmentation‐histomorphometric analysis of graft materials for maxillary sinus floor augmentation. Journal of Oral and Maxillofacial Surgery, 51(8), 857–862. 8393101 10.1016/s0278-2391(10)80103-x

[term2462-bib-0034] Muschler, G. E. , Nakamoto, C. , & Griffith, L. G. (2004). Engineering principles of clinical cell‐based tissue engineering. Journal of Bone and Joint Surgery‐American Volume, 86A(7), 1541–1558. 10.2106/00004623-200407000-00029 15252108

[term2462-bib-0035] National Health Service . (2010). Cleft lip and palate‐treatment [Online]. Available: http://www.nhs.uk/Conditions/Cleft‐lip‐and‐palate/Pages/Treatment.aspx [Accessed Thursday 3rd May 2012].

[term2462-bib-0036] Nauman, E. A. , Satcher, R. L. , Keaveny, T. M. , Halloran, B. P. , & Bikle, D. D. (2001). Osteoblasts respond to pulsatile fluid flow with shortterm increases in PGE(2) but no change in mineralization. Journal of Applied Physiology, 90(5), 1849–1854. 11299276 10.1152/jappl.2001.90.5.1849

[term2462-bib-0037] O'Brien, J. , Wilson, I. , Orton, T. , & Pognan, F. (2000). Investigation of the Alamar Blue (resazurin) fluorescent dye for the assessment of mammalian cell cytotoxicity. European Journal of Biochemistry, 267(17), 5421–5426. 10951200 10.1046/j.1432-1327.2000.01606.x

[term2462-bib-0038] Oliveira, J. M. , Rodrigues, M. T. , Silva, S. S. , Malafaya, P. B. , Gomes, M. E. , Viegas, C. A. , … Reis, R. L. (2006). Novel hydroxyapatite/chitosan bilayered scaffold for osteochondral tissue‐engineering applications: Scaffold design and its performance when seeded with goat bone marrow stromal cells. Biomaterials, 27(36), 6123–6137. 16945410 10.1016/j.biomaterials.2006.07.034

[term2462-bib-0040] Osyczka, A. M. , Damek‐Poprawa, M. , Wojtowicz, A. , & Akintoye, S. O. (2009). Age and skeletal sites affect BMP‐2 responsiveness of human bone marrow stromal cells. Connective Tissue Research, 50(4), 270–277. 19637063 10.1080/03008200902846262 PMC2905683

[term2462-bib-0041] Otsu, N. (1979). A threshold selection method from grey‐level histograms. IEEE Transactions on Systems, Man, and Cybernetics: Systems, 9(1), 62–66.

[term2462-bib-0042] Parkinson, I. H. , Badiei, A. , & Fazzalari, N. L. (2008). Variation in segmentation of bone from micro‐CT imaging: implications for quantitative morphometric analysis. Australasian Physical & Engineering Sciences in Medicine, 31(2), 160–164. 18697709 10.1007/BF03178592

[term2462-bib-0044] Samee, M. , Kasugai, S. , Kondo, H. , Ohya, K. , Shimokawa, H. , & Kuroda, S. (2008). Bone morphogenetic protein‐2 (BMP‐2) and vascular endothelial growth factor (VEGF) transfection to human periosteal cells enhances osteoblast differentiation and bone formation. Journal of Pharmacological Sciences, 108(1), 18–31. 18776714 10.1254/jphs.08036fp

[term2462-bib-0045] Sikavitsas, V. I. , Bancroft, G. N. , Holtorf, H. L. , Jansen, J. A. , & Mikos, A. G. (2003). Mineralized matrix deposition by marrow stromal osteoblasts in 3D perfusion culture increases with increasing fluid shear forces. Proceedings of the National Academy of Sciences of the United States of America, 100(25), 14683–14688. 14657343 10.1073/pnas.2434367100 PMC299759

[term2462-bib-0046] Sittichokechaiwut, A. , Edwards, J. H. , Scutt, A. M. , & Reilly, G. C. (2010). Short bouts of mechanical loading are effective as dexamethasone at inducing matrix production by human bone marrow mesenchymal stem cells. European Cells & Materials, 20, 45–57. 20648425 10.22203/ecm.v020a05

[term2462-bib-0047] Sloan, A. J. , & Waddington, R. J. (2009). Dental pulp stem cells: what, where, how? International Journal of Paediatric Dentistry, 19(1), 61–70. 19120509 10.1111/j.1365-263X.2008.00964.x

[term2462-bib-0048] Sung, H. J. , Meredith, C. , Johnson, C. , & Galis, Z. S. (2004). The effect of scaffold degradation rate on three‐dimensional cell growth and angiogenesis. Biomaterials, 25(26), 5735–5742. 15147819 10.1016/j.biomaterials.2004.01.066

[term2462-bib-0049] Tarng, Y. W. , Casper, M. E. , Fitzsimmons, J. S. , Stone, J. J. , Bekkers, J. , An, K. N. , … Reinholz, G. G. (2010). Directional fluid flow enhances in vitro periosteal tissue growth and chondrogenesis on poly‐epsilon‐caprolactone scaffolds. Journal of Biomedical Materials Research. Part A, 95A(1), 156–163. 10.1002/jbm.a.32830 PMC2928853 20540101

[term2462-bib-0050] Thi, M. M. , Suadicani, S. O. , & Spray, D. C. (2010). Fluid flow‐induced soluble vascular endothelial growth factor isoforms regulate actin adaptation in osteoblasts. Journal of Biological Chemistry, 285(40), 30931–30941. 20682775 10.1074/jbc.M110.114975 PMC2945584

[term2462-bib-0051] Thimm, B. W. , Wechsler, O. , Bohner, M. , Muller, R. , & Hofmann, S. (2013). *In vitro* ceramic scaffold mineralization: comparison between histological and micro‐computed tomographical analysis. Annals of Biomedical Engineering, 41(12), 2666–2675. 23918079 10.1007/s10439-013-0877-4

[term2462-bib-0052] Tormin, A. , Li, O. , Brune, J. C. , Walsh, S. , Schutz, B. , Ehinger, M. , … Scheding, S. (2011). Cd146 expression on primary nonhematopoietic bone marrow stem cells is correlated with *in situ* localization. Blood, 117(19), 5067–5077. 21415267 10.1182/blood-2010-08-304287 PMC3109533

[term2462-bib-0053] Trautvetter, W. , Kaps, C. , Schmelzeisen, R. , Sauerbier, S. , & Sittinger, M. (2011). Tissue‐engineered polymer‐based periosteal bone grafts for maxillary sinus augmentation: Five‐year clinical results. Journal of Oral and Maxillofacial Surgery, 69(11), 2753–2762. 21680073 10.1016/j.joms.2011.02.096

[term2462-bib-0054] Yamaguchi, Y. , Ohno, J. , Sato, A. , Kido, H. , & Fukushima, T. (2014). Mesenchymal stem cell spheroids exhibit enhanced in‐vitro and in‐vivo osteoregenerative potential. BMC Biotechnology, 14, 105. 25479895 10.1186/s12896-014-0105-9 PMC4299781

[term2462-bib-0056] Yuan, L. , Sakamoto, N. , Song, G. , & Sato, M. (2013). High‐level shear stress stimulates endothelial differentiation and VEGF secretion by human mesenchymal stem cells. Cellular and Molecular Bioengineering, 6, 220–229.

[term2462-bib-0057] Zargarian, S. S. , & Haddadi‐Asl, V. (2010). A nanofibrous composite scaffold of PCL/hydroxyapatite‐chitosan/PVA prepared by electrospinning. Iranian Polymer Journal, 19(6), 457–468.

[term2462-bib-0058] Zhou, Y. , Guan, X. , Zhu, Z. , Gao, S. , Zhang, C. , Li, C. , … Yu, H. (2011). Osteogenic differentitation of bone marrow‐derived mesenchymal stromal cells on bone‐derived scaffolds: Effect of microvibration and role ERK1/2 activation. European Cells & Materials, 22, 12–25. 21732279 10.22203/ecm.v022a02

