# Supplementary figures and images for: A simple rocker‐induced mechanical stimulus upregulates mineralization by human osteoprogenitor cells in fibrous scaffolds
Source: J Tissue Eng Regen Med. 2017 Aug 9;12(2):370–81. doi: 10.1002/term.2462 (PMC5836908; doi:10.1002/term.2462)

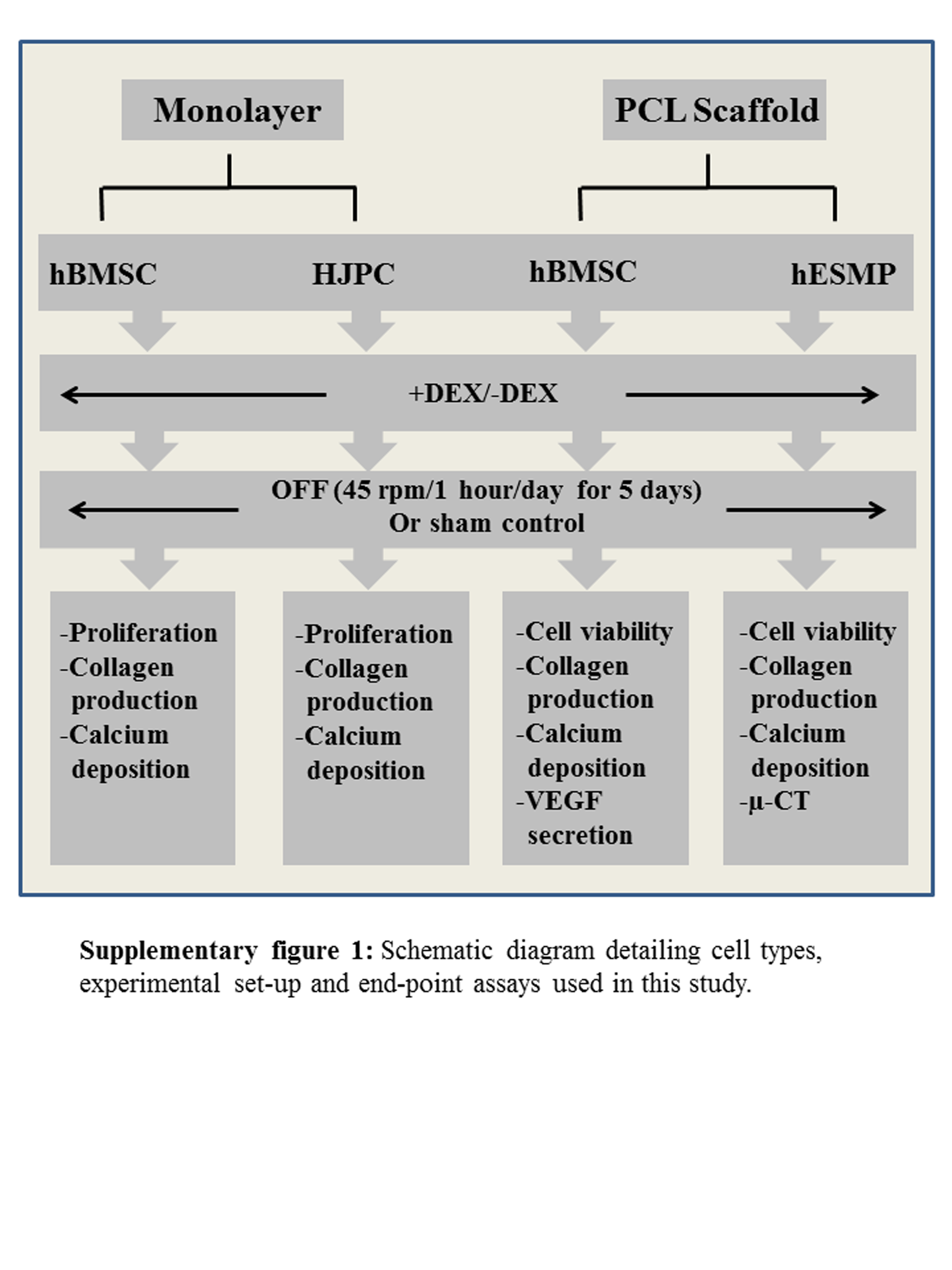

Supplement: Supplementary file 1 — FIGURE S1. Schematic diagram detailing cell types, experimental set‐up and end‐point assays used in this study. [file TERM-12-370-s001.tif]

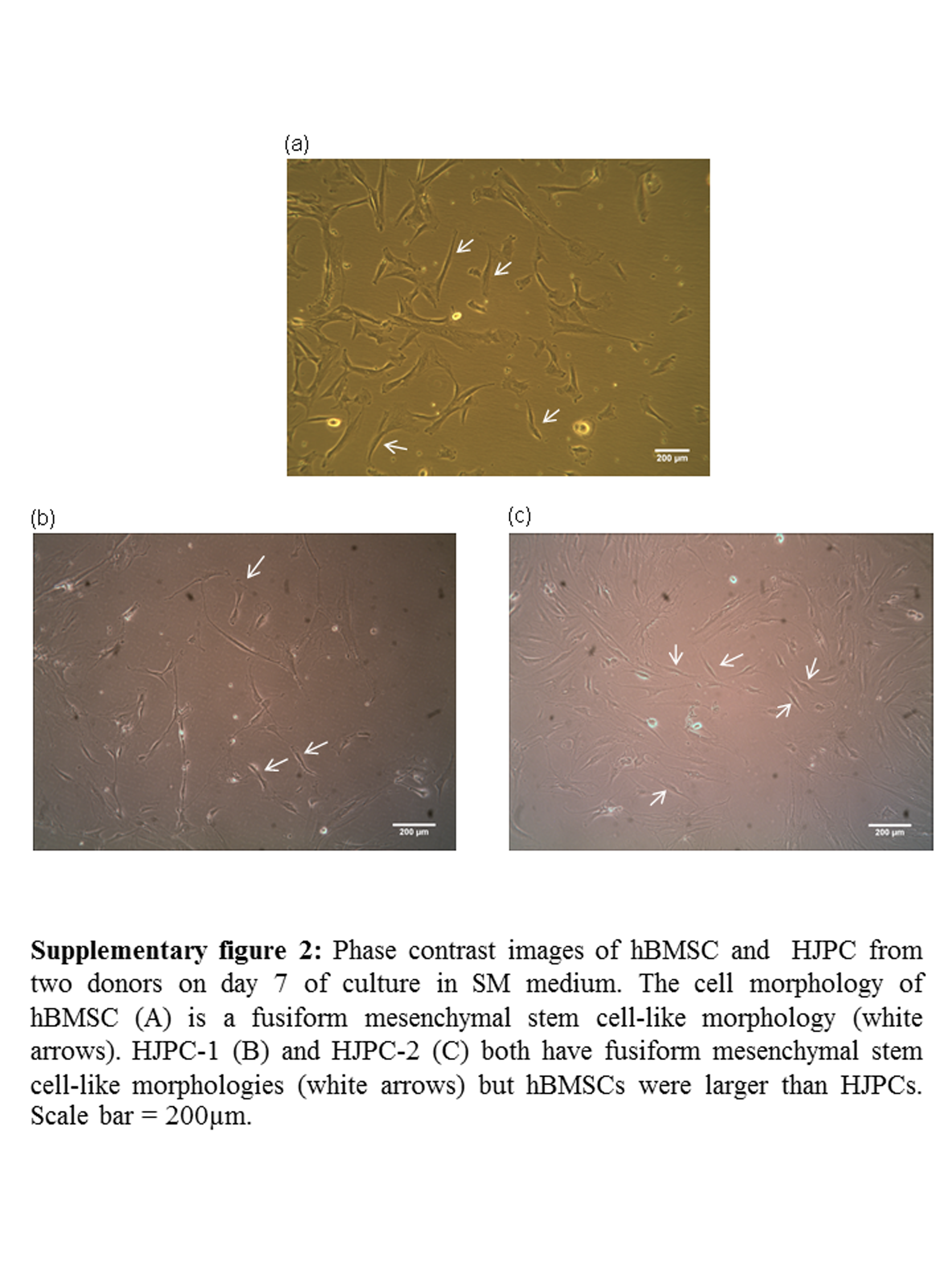

Supplement: Supplementary file 2 — FIGURE S2. Phase contrast images of hBMSC and HJPC from two donors on day 7 of culture in SM medium. The cell morphology of hBMSC (a) is a fusiform mesenchymal cell0like morphology. HJPC‐1 (b) and HJPC‐2 (c) both have fusiform mesenchymal stem cell‐like morphologies (white arrows) but hBMSCs were larger the HJPCs. Scale bar = 200 μm. [file TERM-12-370-s002.tif]
