# Supplementary material for: A simple rocker‐induced mechanical stimulus upregulates mineralization by human osteoprogenitor cells in fibrous scaffolds
Source: J Tissue Eng Regen Med. 2017 Aug 9;12(2):370–81. doi: 10.1002/term.2462 (PMC5836908; doi:10.1002/term.2462)
Supplement: Supplementary file 3 — Table S1: Surface antigen expression patterns of hESMP, HJPC‐1, HJPC‐2, hBMSC‐1, hBMSC‐2, and hBMSC‐3were measured using flow‐assisted cell sorting. [file TERM-12-370-s003.docx]

| **Cell types** | **CD146** | **CD105** | **CD90** | **CD45** |
| --- | --- | --- | --- | --- |
| hESMPs (cell line) | **96.7** | **99.9** | **86.1** | **0.8** |
| HJPC-1 | **1.0** | **97.3** | **68.5** | **0.5** |
| HJPC-2 | **8.1** | **99.3** | **97.9** | **5.0** |
| hBMSC-1 | **94.5** | **99.6** | **97.8** | **3.3** |
| hBMSC-2 | **66.7** | **99.9** | **99.7** | **0.8** |
| hBMSC-3 | **95.5** | **100.0** | **100.0** | **10.3** |

**Supplementary data 3:** Surface antigen expression patterns of hESMP, HJPC-1, HJPC-2, hBMSC-1, hBMSC-2, and hBMSC-3were measured using FACS.
